# Editorial: Anti-HLA DSA and Beyond: Deciphering the Immunological Mechanisms Driving Chronic Rejection

**DOI:** 10.3389/ti.2026.16410

**Published:** 2026-02-27

**Authors:** Federica Casiraghi, Maarten Naesens, Aravind Cherukuri, Sandra Lindstedt, Candice Roufosse, Olivier Thaunat

**Affiliations:** 1 Department of Molecular Medicine, Istituto di Ricerche Farmacologiche Mario Negri IRCCS, Bergamo, Italy; 2 Department of Microbiology, Immunology and Transplantation, KU Leuven, Leuven, Belgium; 3 Thomas E. Starzl Transplantation Institute, University of Pittsburgh, Pittsburgh, PA, United States; 4 Department of Cardiothoracic Surgery and Transplantation, Lund University Hospital, Lund, Sweden; 5 Department Immunology and Inflammation, Imperial College London, London, United Kingdom; 6 Department of Nephrology, Transplantation and Clinical Immunology, Hospices Civils de Lyon, Groupement Hospitalier Centre, Lyon, France; 7 Lyon-Est Medical Faculty, Claude Bernard University (Lyon 1), Lyon, France

**Keywords:** donor-specific antibodies, innate immune cells, microvascular injury, natural killer cells, non-HLA antibodies

## From Anti-HLA DSA to Integrated Models of Chronic Rejection

Solid organ transplantation remains the optimal therapy for patients with end-stage organ failure. Yet, despite major advances in therapeutic immunosuppression over the past two decades, chronic immune-mediated injury of allograft continues to be a leading cause of late graft failure.

**FIGURE 1:**
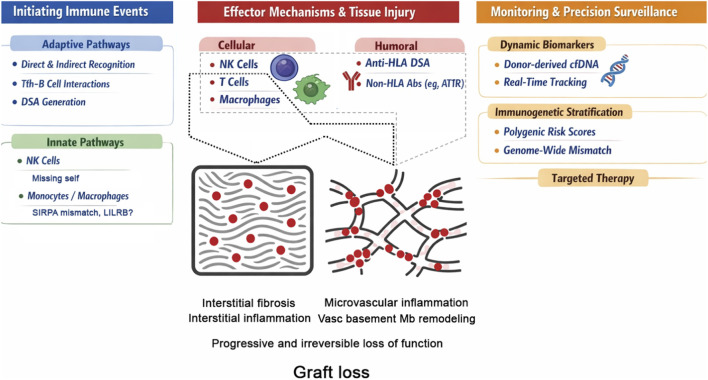
Integrated Models of Chronic Rejection. Chronic rejection results from multiple innate and adaptive allorecognition pathways that activate effector mechanisms, causing interstitial and microvascular inflammation and progressive fibrosis. Dynamic biomarkers, including donor-specific HLA antibodies (DSA) and donor-derived cell-free DNA (dd-cfDNA), may early detect graft injury, guiding the biopsy. Integrating immunologic, histologic, and genetic data should enable mechanism-informed monitoring and targeted interventions before irreversible damage occurs.

Anti–donor-specific HLA antibodies (DSA) have long occupied a central place in this process, consistent with the strong association between the presence of circulating DSA, microvascular inflammation in graft biopsy and poor long-term outcomes. However, several observations challenge a strictly DSA-centric model: chronic microvascular rejection–like lesions may arise in the absence of detectable circulating DSA; pathological patterns vary widely across organs and patients; and graft injury may progress even when humoral activity appears controlled. Together, these findings support a more integrated framework in which multiple pathways of allorecognition and immune effector mechanisms—adaptive and innate—converge on the graft microvasculature, driving endothelial injury, remodeling, and fibrosis (Figure 1).

This Special Issue follows this conceptual trajectory, from allorecognition to chronic tissue damage, highlighting how adaptive and innate immunity intersect to shape late transplant outcomes.

## Progress in Elucidating the Mechanisms Underlying Allorecognition

### Indirect Allorecognition, Tfh–B Cell Interactions, and the Maintenance of Pathogenic Antibody Responses

Basu et al. review the pivotal role of the indirect pathway of allorecognition—presentation of donor-derived peptides by recipient antigen-presenting cells in the context of self HLA—in sustaining helper T-cell responses, DSA generation, and chronic rejection. They detail how donor-specific B cells present processed alloantigen to CD4^+^ T cells, frequently adopting a T follicular helper phenotype that maintains germinal center reactions, affinity maturation, and pathogenic antibody production. Importantly, the review also outlines endogenous regulatory mechanisms, describing how distinct regulatory T- and B-cell subsets can suppress cytokine release, plasmablast differentiation, antibody secretion, and germinal center maturation. These insights identify specific cellular and molecular targets for future immunoregulatory or cell-based therapeutic strategies.

### Expanding Allorecognition Pathways and Innate Contributions

Charmetant et al. revisit the classical direct and indirect pathways of allorecognition by incorporating mechanisms that better explain persistent alloimmunity. They emphasize the semi-direct pathway—where recipient antigen-presenting cells acquire intact donor MHC molecules from graft cells—and introduce the concept of the “three-cell cluster” as a functional refinement sustaining long-term T-cell activation. The review also discusses the inverted direct pathway, whereby donor CD4^+^ T cells provide help to recipient B cells, potentially supporting early DSA generation. Beyond adaptive immunity, the authors highlight antibody-independent roles of NK cells and describe how innate myeloid cells contribute to non-self recognition and initiation of adaptive responses, positioning innate immunity as a potential trigger and amplifier of late graft injury.

### Innate Non-Self Recognition by Myeloid Cells

Focusing specifically on innate discrimination mechanisms, Palvair et al. examine non-self recognition by monocytes and macrophages, proposing leukocyte immunoglobulin-like receptors (LILRs)—particularly LILRB3—as candidate mediators of allograft injury. Building on murine data on paired immunoglobulin receptor–dependent recognition, they describe LILRs as human orthologs capable of modulating myeloid activation in transplantation. The review summarizes evidence that LILRB3 may bind HLA class I molecules and interact with complement fragments and other ligands, and suggests that receptor polymorphism and the balance between inhibitory and activating counterparts (e.g., LILRA6) may polarize myeloid responses toward inflammatory or regulatory phenotypes. This work provides a conceptual bridge between innate recognition and chronic graft injury beyond a purely antibody-centered paradigm.

## Effector Mechanisms Driving Allograft Injury

### Beyond HLA: Mapping the Non-HLA Antibody Landscape

Addressing antibody-mediated mechanisms beyond HLA, Schmidt et al. present an original study profiling 60 non-HLA antibodies in serial serum samples from 77 pediatric kidney transplant recipients. Substantial pre-transplant reactivity was observed, with more than half of patients displaying over 15 positive antibodies, and overall antibody burden remained relatively stable early after transplantation. While cumulative non-HLA antibody profiles did not clearly predict late antibody-mediated rejection (AMR), specific targets emerged as potential signals, including SNRPB2 pre-transplant and ACTIN/CGB5 at 1–2 years post-transplant. These findings suggest that selected non-HLA specificities—rather than overall antibody load—may hold clinical relevance, pending independent validation.

### AT1R Antibodies and Endothelial Activation

Martin et al. focus on antibodies against the angiotensin II type 1 receptor (AT1R), among the most extensively studied non-HLA specificities associated with AMR-like phenotypes. They describe how AT1R antibodies can function as receptor agonists, sustaining endothelial signaling through pathways including β-arrestin and mTOR, thereby promoting vascular inflammation and impaired repair, often with limited complement deposition. Clinically, the review synthesizes heterogeneous observational evidence and underscores key challenges to implementation, including assay standardization, threshold definition, and prospective validation. The authors conclude with a pragmatic message: AT1R antibodies may be relevant in selected contexts, but routine testing requires stronger and more consistent evidence.

### NK Cells as Central Effectors of Chronic Vascular Injury

Chambon et al. position NK cells as central drivers of chronic vascular rejection, acting both downstream of antibodies and through antibody-independent mechanisms. They organize NK activation into three major pathways: missing-self recognition due to reduced inhibitory HLA class I signaling, induced-self recognition of stress ligands, and antibody-dependent cellular cytotoxicity via FcγRIIIA engagement. Importantly, the review emphasizes that NK cells require priming to become fully pathogenic and can, in experimental models, recruit additional effectors such as T cells and macrophages through cytokines including IFN-γ, thereby sustaining inflammatory circuits that promote chronic vascular remodeling.

## Mechanistic Insights From Allograft Biopsies

### Tissue-Level Evidence of NK Cell Involvement in ABMR

Providing biopsy-level support for NK cell involvement, Diebold et al. combine CD16 and T-bet immunostaining with histologic and molecular analyses. NK cell infiltration, particularly within peritubular capillaries, correlates strongly with microvascular inflammation (Banff glomerulitis plus peritubular capillaritis scores), while showing little association with tubulitis or chronic tubulointerstitial lesions. NK burden also aligns with Molecular Microscope Diagnostic System–derived AMR classifiers and NK transcript burden scores, but not with T cell–mediated rejection probability. The authors discuss timing-dependent mechanisms and propose that ADCC may be especially relevant in late AMR, linking tissue patterns to effector biology.

### Rethinking Rejection Histology Through a Mechanistic Lens

Terinte-Balcan et al. reframe kidney transplant rejection histology by moving beyond a strict TCMR-versus-AMR dichotomy and examining which immune cells dominate specific lesion patterns. Using Banff lesions as a reference, they illustrate how multiplex immunofluorescence and bulk, single-cell, and spatial transcriptomics are redefining inflammatory landscapes across active AMR, chronic active AMR—including transplant glomerulopathy—and acute and chronic active TCMR. A key message is the marked heterogeneity within diagnostic categories and the increasing recognition of antibody-independent and non-HLA–related processes shaping histologic phenotypes and prognosis.

### Microvascular Inflammation Beyond Antibody-Mediated Rejection

Varol et al. focus on microvascular inflammation—glomerulitis and peritubular capillaritis—traditionally considered a hallmark of AMR, and challenge its exclusivity. Through a systematic review and re-analysis of a local biopsy cohort, they demonstrate that microvascular inflammation can also occur in acute TCMR, albeit with lower frequency and severity. They discuss how these findings complicate Banff-based categorization and relate them to evolving Banff concepts, including DSA- and C4d-negative microvascular inflammation phenotypes and potential contributions of non-HLA antibodies and innate activation.

### Transplant Glomerulopathy as a Convergent Phenotype

Using transplant glomerulopathy as a paradigmatic lesion of chronic rejection, Chutani et al. explicitly question a one-to-one equivalence with DSA-mediated mechanisms. After reviewing defining morphologic features and prognostic implications, they broaden interpretation by discussing DSA-negative transplant glomerulopathy and multiple contributors converging on endothelial injury, including non-HLA antibodies, complement-related pathways, cell-mediated mechanisms, and podocyte stress. The review emphasizes transplant glomerulopathy as a histologic endpoint integrating diverse upstream immune processes.

## From Injury Biomarkers to Precision Surveillance

### Donor-Derived Cell-Free DNA for Dynamic Monitoring

Akifova et al. discuss donor-derived cell-free DNA (dd-cfDNA) as a practical tool to reduce diagnostic uncertainty in AMR, particularly in DSA-positive recipients. Given the limited sensitivity of creatinine and proteinuria for subclinical injury and the frequent absence of histologic AMR in for-cause biopsies, dd-cfDNA is presented as a dynamic injury-linked biomarker with a short half-life. The review summarizes technical considerations and associations with microvascular inflammation and molecular AMR signatures, while highlighting the need to prospectively define appropriate contexts of use.

In a brief research report, the same group Osmanodja et al. presents longitudinal dd-cfDNA monitoring in two sensitized kidney transplant recipients with biopsy-proven AMR treated with daratumumab. In both cases, dd-cfDNA trajectories closely mirrored histologic activity, decreasing with clinical improvement and aligning with post-treatment biopsy findings. Although limited by sample size, these cases provide proof-of-concept for integrating dd-cfDNA into treatment-response monitoring and follow-up strategies in difficult-to-treat AMR.

### Toward Immunogenetically Informed Surveillance

Finally, Helanterä et al. extend monitoring strategies into a precision-medicine framework by examining how immunogenetics may stratify risk and guide post-transplant surveillance. They summarize emerging evidence linking polygenic risk scores and genome-wide mismatch concepts to post-transplant phenotypes, while acknowledging that current predictive gains remain modest. Importantly, the authors outline a translational perspective in which validated genetic signals could support individualized immunosuppression and targeted monitoring in high-risk subsets.

## Conclusion

Collectively, these contributions portray chronic rejection as the result of layered and interacting immune pathways, in which humoral, cellular, and innate mechanisms converge on the graft microvasculature to drive progressive remodeling and fibrosis. Improving long-term graft survival will depend on mechanism-informed diagnostics and surveillance, enabling earlier intervention and paving the way for targeted therapies capable of interrupting chronic injury before it becomes irreversible.

